# Silibinin’s Effects against Methotrexate-Induced Hepatotoxicity in Adjuvant-Induced Arthritis Rat Model

**DOI:** 10.3390/ph17040431

**Published:** 2024-03-28

**Authors:** Ghada Khawaja, Youmna El-Orfali

**Affiliations:** 1 Department of Biological Sciences, Faculty of Science, Beirut Arab University, Beirut 11-5020, Lebanon; ye25@aub.edu.lb; 2 Department of Experimental Pathology, Immunology and Microbiology, Faculty of Medicine, American University of Beirut, Beirut 11-0236, Lebanon

**Keywords:** silibinin (SIL), arthritis experimental model, antioxidant, anti-arthritic, hepatoprotective

## Abstract

Methotrexate (MTX) is the first drug of choice to treat several diseases, including rheumatoid arthritis. However, its administration is accompanied by severe side effects, most commonly hepatotoxicity. Hence, alternative therapies with a lower toxicity and fewer side effects are needed. This study aimed to investigate the antioxidant and hepatoprotective effects of silibinin (SIL, natural agent) against MTX-induced hepatotoxicity in an adjuvant-induced arthritis (AIA) rat model. Arthritic rats were treated with SIL (100 mg/kg) and/or methotrexate (2 mg/kg). Non-arthritic rats, arthritic untreated rats, and arthritic rats who received the vehicle were followed in parallel. SIL alleviated the systemic consequences of arthritis by restoring lost weight, decreasing the erythrocyte sedimentation rate, and ameliorating joint damage, which was evident both micro- and macroscopically. Additionally, SIL prevented the histopathological alterations in the liver and significantly reduced the liver damage caused by MTX and AIA, as shown by a decrease in the markers of liver damage (ALT and AST). Furthermore, SIL relieved the oxidative stress induced by AIA and MTX in liver tissue by decreasing the lipid peroxidation (MDA) levels and enhancing the antioxidant defense system (GSH levels; catalase and superoxide dismutase (SOD) activities). In conclusion, our results suggest that SIL is a potent antioxidant and hepatoprotective agent in arthritic rats. It markedly attenuated the progression and severity of the arthritic disease and eased the oxidative stress in liver tissue by improving the pro-oxidant/antioxidant balance.

## 1. Introduction

Rheumatoid arthritis (RA) is a chronic inflammatory autoimmune disorder where synovial joints and other tissues are damaged by the immune system [1]. RA is marked by chronic pain and joint destruction through the persistent and progressive synovitis of peripheral joints, which eventually leads to cartilage and subchondral bone damage. RA is the most common disease of the inflammatory joint, affecting up to 0.5–1% of the world’s population. The autoimmune response mediates local synovial inflammation and cellular infiltration, which ultimately results in tissue damage [2].

An imbalance between pro- and anti-inflammatory cytokine activities favors the induction of autoimmunity, chronic inflammation, and consequently, joint damage [3]. In RA, joint macrophages secrete pro-inflammatory cytokines and growth factors such as IL-1, IL-2, IL-6, and tumor necrosis factor alpha (TNF-α). These cytokines cause local joint damage due to increased metalloproteinase production (MMP) and osteoclast activation. IL-1, IL-6, and TNF-α also leak into the bloodstream, resulting in systemic inflammation that affects many organs, including the bone (anemia, thrombocytosis, and osteoporosis), brain (fever, fatigue, and depression), and liver (acute-phase response) [4,5].

The overproduction of pro-inflammatory cytokines stimulates neutrophils. This activation, in turn, leads to the secretion of reactive oxygen species (ROS) by macrophages in the synovial fluid, which causes tissue injury [6]. TNF-α and IL-1 activate Kupffer cells (liver macrophages) that undergo an oxidative burst after activation, which produces highly toxic ROS to kill the invading pathogens [7]. The NADPH oxidase mechanism mediates this oxidative explosion, resulting in a marked increase in oxygen consumption and superoxide development (O_2_^−^•).

The increased ROS content in arthritic rat livers appears to be the product of both a stimulated pro-oxidant mechanism and defective antioxidant protection, such as reduced glutathione (GSH) and catalase (CAT). Therefore, RA is not only a systemic disease characterized by systemic inflammation, but is also accompanied by a systemic redox imbalance [8].

There is a consensus on a cure for RA; thus, the main goals of therapy are to relieve the disease’s symptoms [9]. MTX is considered popular among disease-modifying anti-rheumatic drugs (DMARDs) and is considered the first medical treatment option for RA worldwide [10]. MTX is typically given to patients with RA once a week, with the doses varying from 7.5 to 25 mg/day. Following absorption, 10% of the MTX is converted into 7-hydroxymethotrexate in the liver. A portion of MTX and 7-hydroxymethotrexate is metabolized into polyglutamate derivatives once they are taken up by cells. MTX polyglutamates (MTXGlus) are retained in tissues for long periods of time, including in the liver and erythrocytes [11]. MTX uses a number of cellular pathways to project its actions [12]. Nevertheless, hepatotoxicity has been associated with the prolonged administration of this agent [13].

As natural compounds are less toxic and cost effective, these compounds are gaining researchers’ interest. One of these natural agents is silibinin (SIL). SIL/silybin is the main active component of silymarin (SM), a mixture of milk-thistle-derived flavonolignans (Silybum marianum) [14]. In general, SM is used to treat liver disorders of different etiologies, and the extracts of milk thistle were used as traditional herbal remedies (“liver tonics”) [15]. SIL is, therefore, best known for its antioxidant and chemical effects on the liver [16]. SIL acts in four different ways [15]: as an antioxidant, scavenger, and regulator of glutathione intracellular content [17]; as a cell membrane stabilizer and regulator of the permeability of hepatotoxic agents [18]; as a promoter of ribosomal RNA synthesis, stimulating liver regeneration [19]; and as an inhibitor of stellate hepatocyte transformation into myofibroblasts, which is the mechanism mainly responsible for the deposition of collagen fibers that leads to cirrhosis [20].

Adjuvant-induced arthritis (AIA) is an experimental model that has been used widely for the preclinical testing of numerous anti-arthritic agents [21,22]. This study aimed to investigate the anti-inflammatory and anti-arthritic effects of SIL in comparison with MTX and to assess the antioxidant and hepatoprotective effects against MTX-induced hepatotoxicity in an AIA rat model.

## 2. Results

### 2.1. Effect of SIL on Disease Severity in AIA Rat Model

#### 2.1.1. Body Weight

**Figure 1:**
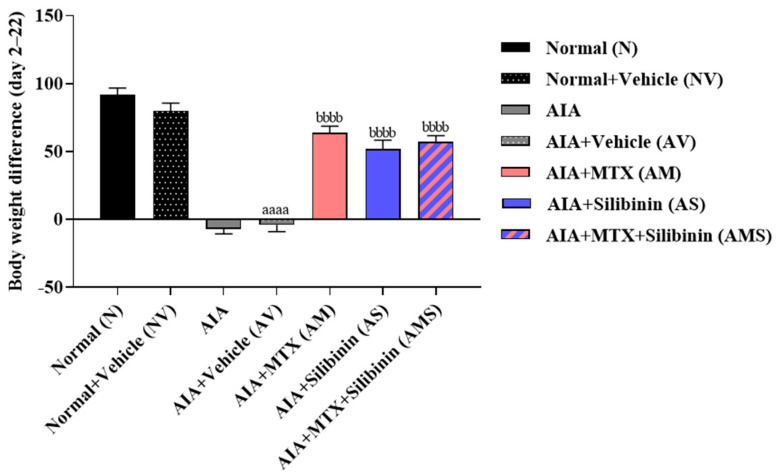
Effect of SIL on body weight of arthritic rats. Mean body weight in rat groups was monitored over a period of 23 days. Difference in body weight between day 22 and day 2 was calculated. All values are presented as mean ± SD of 8 rats per group. aaaa: *p* < 0.0001 compared to normal group (N); bbbb: *p* < 0.0001 compared to arthritic vehicle-treated group (AV). AIA: adjuvant-induced arthritis; MTX: methotrexate.

During a study, it was observed that untreated rats suffering from arthritis experienced a significant drop in body weight compared to healthy rats. This suggested the severity of their arthritic condition. However, when arthritic rats were treated with 2 mg/kg of MTX (AM), 100 mg/kg of SIL (AS), or both (AMS), they showed a marked increase in body weight from day 2 to day 22 compared to untreated arthritic rats (AIA and AV). There were no significant statistical differences between the treated groups and the healthy groups (Figure 1).

#### 2.1.2. Macroscopic Scoring of the Injected Paw

**Figure 2:**
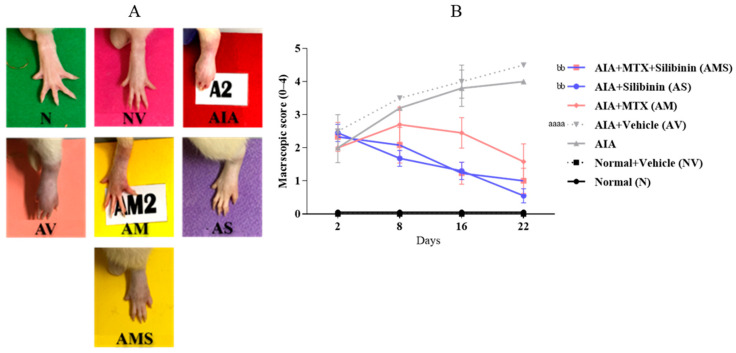
Effect of SIL on macroscopic score of arthritic rats. (**A**) Pictures of injected rat paw on day 16. Scores of representative rat in each group are mentioned between brackets: (N) normal (0), (NV) normal + vehicle (0), (AIA) arthritic (4), (AV) AIA + vehicle (4), (AM) AIA + MTX (1), (AS) AIA + SIL (<1), and (AMS) AIA + MTX + SIL (1). (**B**) Mean macroscopic score (0–4) in rat groups was monitored over a period of 23 days. All values are presented as mean ± SD of 8 rats per group. aaaa: *p* < 0.0001 compared with normal group on day 22; bb: <0.01 compared to arthritic vehicle-treated group (AV) on day 22. AIA: adjuvant-induced arthritis; MTX: methotrexate.

The arthritic untreated rats showed a progressive increase in their macroscopic score on day 22, with a peak score of 4. The rats treated with 100 mg/kg of SIL (AS), 2 mg/kg of MTX (AM), or SIL + MTX (AMS) showed a clear decrease in paw inflammation and edema compared to the arthritic untreated model group rats and subsequently showed a decrease in their macroscopic rating (Figure 2A,B).

#### 2.1.3. Histological Examination of the Tibiotarsal Joint

**Figure 3:**
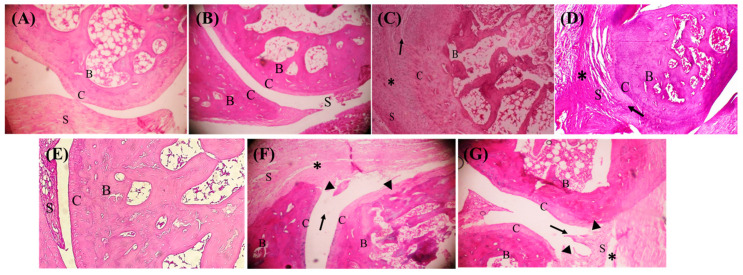
Effect of SIL on tibiotarsal joint in ankle stained with H&E. (**A**,**B**) Normal rats with or without SIL vehicle exhibited normal ankles (grade 0). (**C**,**D**) Arthritic rats with or without SIL vehicle showed severe ankle alterations (grade 3). (**E**) Arthritic rats treated with MTX displayed moderate ankle alterations (grade 2). (**F**,**G**) Arthritic rats treated with SIL +/− MTX revealed mild ankle alterations (grade 2). Cellular infiltration (*), synovial hyperplasia (↑), pannus formation (►); B: bone, C: cartilage, S: synovium.

A notable distinction between the groups was found by the histological examination of the tibiotarsal joint. The normal groups (with or without the SIL vehicle) showed normal tibiotarsal joint features in the ankle, with no joint inflammation and an intact synovium (grade 0). There was no sign of pannus growth forming or penetrating the cartilage or bone, nor any cellular infiltration extending from the synovium to the capsule (grade 0). However, the arthritic groups (with or without the SIL vehicle) exhibited extensive synovial hyperplasia in their tibiotarsal joint (grade 3). Furthermore, there was widespread and clear evidence of invasive granulation tissue production or pannus formation. In contrast, the arthritic groups treated with 2 mg/kg of MTX, 100 mg/kg of SIL, or SIL + MTX demonstrated reduced pathological joint alterations. There was a reduction in synovium hyperplasia in two to four layers of synoviocytes, with a severe cellular invasion, but no aggregate development (grade 2). Pannus formation, however, was moderate for the MTX treatment, but mild for the SIL and the SIL + MTX treatment (Figure 3).

#### 2.1.4. ESR: Erythrocyte Sedimentation Rate Determination

**Figure 4:**
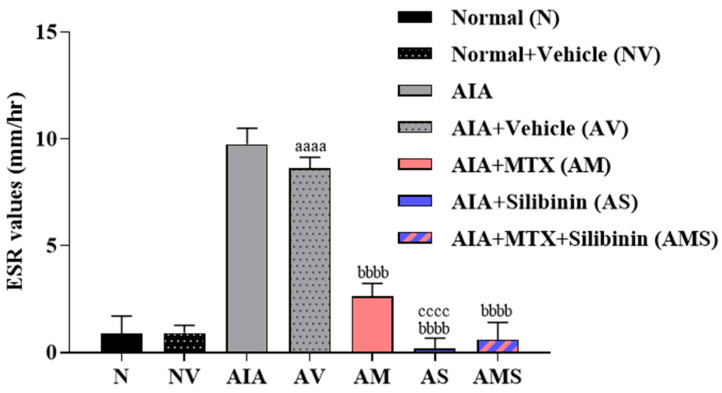
Effect of SIL on ESR of arthritic rats. Mean ESR (mm/hr) in rat groups was measured on day 23. All values are presented as mean ± SD of 8 rats per group. aaaa: *p* < 0.0001 compared to normal group (N), bbbb: *p* < 0.0001 compared to arthritic vehicle-treated group (AV), cccc: *p* < 0.0001 compared to positive control group (AM). AIA: adjuvant-induced arthritis; MTX: methotrexate.

On day 23, an increased ESR value was demonstrated in the arthritic untreated model group. However, the arthritic groups treated with 2 mg/kg of MTX, 100 mg/kg of SIL, or SIL + MTX showed a decrease of approximately 3-, 4.5-, and 4-fold compared to the untreated arthritic group. The ESR levels dropped significantly compared to the positive control group (AIA + MTX) in the arthritic groups treated with SIL or SIL + MTX. There were no significant statistical differences on day 23 between the treated groups and the normal groups (Figure 4).

### 2.2. Effect of SIL on Liver Injury Induced in AIA Rat Model and MTX Treatment

#### 2.2.1. Histological Examination of Liver Sections

**Figure 5:**
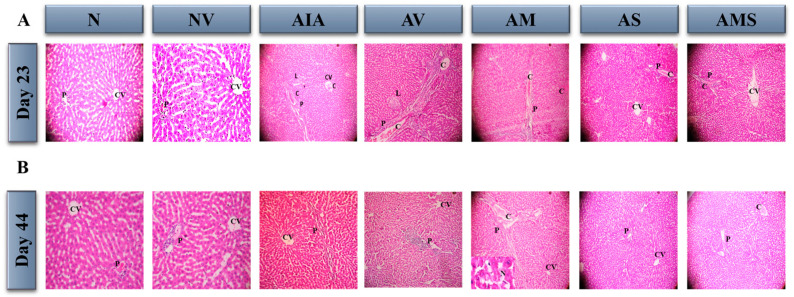
Effect of SIL on histopathological changes in liver of arthritic rats. Photomicrographs of liver sections were stained by H&E (×100). (**A**) Day 23. Liver sections of normal (N) and normal + vehicle (NV) rats showed well-preserved architecture and cytoplasm. Arthritic untreated rats (AIA), arthritic rats treated with vehicle (AV), and arthritic rats treated with positive control MTX (AM) showed severe degeneration in hepatocytes and cytoplasm. Arthritic rats treated with SIL (AS) showed normal hepatocytes and cytoplasm. Rats treated with combination of AIA + MTX + SIL (AMS) showed mild degeneration in hepatocytes and cytoplasm. (**B**) Day 44. Liver sections of normal (N) and normal + vehicle (NV) rats showed well-preserved architecture and cytoplasm. Arthritic untreated rats (AIA) and arthritic rats treated with vehicle (AV) showed severe degeneration in hepatocytes and cytoplasm. Arthritic rats treated with SIL (AS) showed normal hepatocytes and cytoplasm. Arthritic rats treated with MTX (AM) showed severe hepatocyte degeneration and inflammation (+2) plus necrosis. Rats treated with combination of AIA + MTX + SIL (AMS) showed mild degeneration in hepatocytes and cytoplasm. P: portal inflammation, L: lobular inflammation, CV: central vein, C: congested portal tract and lobular part. AIA: adjuvant-induced arthritis.

The normal rats exhibited a normal histological structure of the central vein with normal surrounding hepatocytes and a normal portal tract on both endpoints, day 23 and day 44. On day 23, the arthritic untreated (AIA) and arthritic vehicle-treated rats (AV) showed severe degeneration in their hepatocytes and cytoplasm, with lymphocyte infiltration (+2) and central congestion in the portal and lobular tracts. On day 44, there was no lobular inflammation and the inflammation decreased in severity (+1). Nonetheless, on day 23 and 44, the positive control group, comprising arthritic rats treated with 2 mg/kg of MTX (AM), displayed significant hepatocyte degeneration and a cytoplasm with lymphocyte infiltration in the portal and lobular tracts ((+2) and (+3)). This was accompanied by necrosis on day 44. Importantly, on both days 23 and 44, the arthritic rats treated with 100 mg/kg of SIL (AS) demonstrated a normal histological structure of the central vein with normal surrounding hepatocytes and a normal portal tract. In addition, the AIA + MTX + SIL (AMS) treatment of arthritic rats resulted in mild degeneration in the hepatocytes and cytoplasm, with lymphocyte infiltration in the portal tract (+1) and a congested portal tract and central vein on days 23 and 44, respectively (Figure 5A,B).

#### 2.2.2. Serum ALT and AST Levels

**Figure 6:**
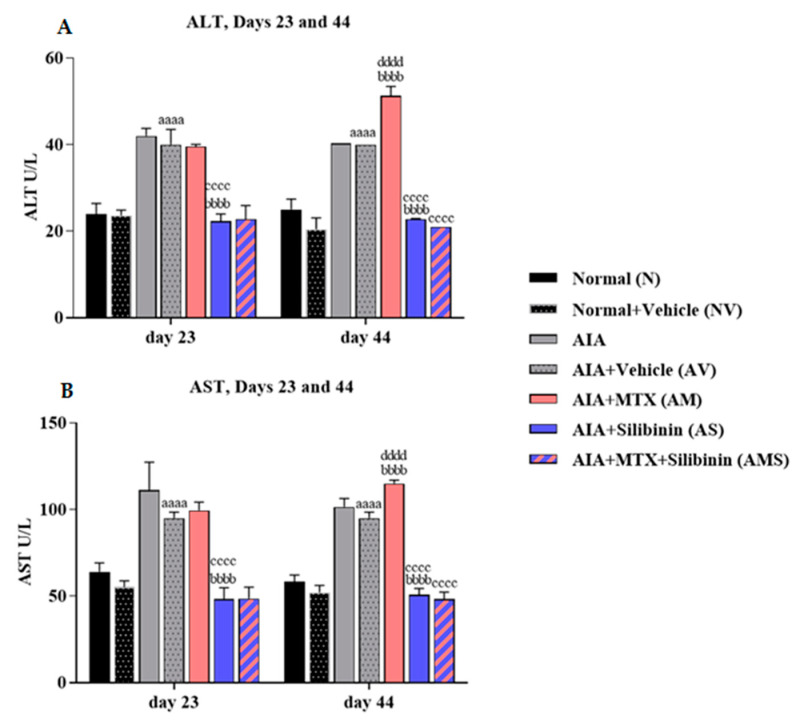
Effect of SIL on liver function enzymes in arthritic rats on days 23 (n = 4) and 44 (n = 4). (**A**) Mean ALT values (U/L). (**B**) Mean AST values (U/L). aaaa: *p* < 0.0001 compared to normal group (N), bbbb: *p* < 0.0001 compared to arthritic vehicle-treated group (AV), cccc: *p* < 0.0001 compared to positive control group (AM), dddd: *p* < 0.0001 compared to day 23. AIA: adjuvant-induced arthritis; MTX: methotrexate.

The serum ALT and AST levels in the AV group were significantly higher on both days 23 and 44 relative to the NV group. On day 44, the AM rats showed a significant improvement in the rate of ALT and AST relative to the AV group. However, the arthritic treatment group (AS) showed a significant decline in its ALT and AST levels on both days 23 and 44 compared to the AV and AM groups. The combination group (AMS) showed a significant reduction in its levels of ALT and AST compared to the positive control group (AM). The AIA + MTX group (AM) showed a significant increase in its ALT and AST levels when comparing the levels on day 44 with day 23 (Figure 6A,B).

### 2.3. Effect of SIL on Oxidative Stress and Antioxidant Defense System in the Liver of AIA Rats

#### 2.3.1. Malondialdehyde Level

**Figure 7:**
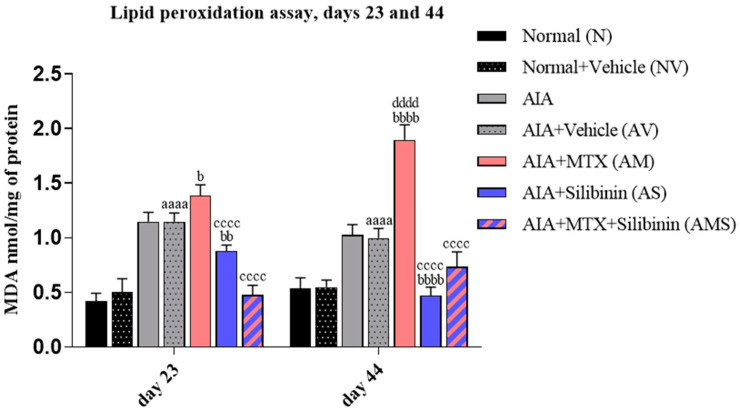
Effect of SIL on oxidative stress in arthritic rats on days 23 (n = 4) and 44 (n = 4). Mean MDA values (nmol/mg). aaaa: *p* < 0.0001 compared to normal group (N), b: <0.05, bb: <0.01, and bbbb: *p* < 0.0001 compared to arthritic vehicle-treated group (AV), cccc: *p* < 0.0001 compared to positive control group (AM), dddd: *p* < 0.0001 compared to day 23. AIA: adjuvant-induced arthritis; MTX: methotrexate.

Compared to the NV group on both days 23 and 44, the MDA levels were significantly elevated in the AV group. The AIA + MTX group (AM) showed a significant rise in its MDA levels on day 44 compared to the AV group. However, the arthritic group treated with SIL (AS) showed a significant reduction in its MDA levels compared to the AV and AM groups on both days 23 and 44, without any significant difference when compared to the NV group on day 44 (Figure 7). The combination group (AMS) showed a significant decrease in its MDA levels compared to the positive control group (AM) on day 44. The AM rats showed a substantial increase in their MDA levels on day 44 when compared to day 23 to 44.

#### 2.3.2. Superoxide Dismutase and Catalase Activities and Reduced Glutathione Levels

**Figure 8:**
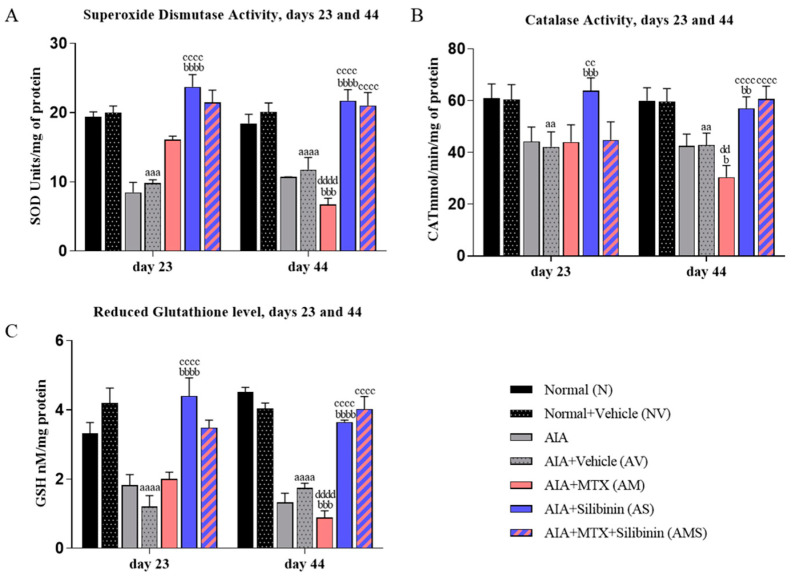
Effect of SIL on antioxidant parameters in arthritic rats on days 23 (n = 4) and 44 (n = 4). (**A**) Mean of superoxide anion activity (U/mg). (**B**) Mean of catalase activity (mmol/min/mg). (**C**) Mean of reduced glutathione levels (nM/mg). aa: *p* < 0.01; aaa: *p* < 0.001 compared to normal group (N); aaaa: *p* < 0 compared to normal group (N); b: <0.05, bb: <0.01, bbb: *p* < 0.001, and bbbb: <0.0001 compared to arthritic vehicle-treated group (AV); cc: <0.01, and cccc: *p* < 0.0001 compared to positive control group (AM); dd: *p* < 0.01 and dddd: *p* < 0.0001 compared to day 23. AIA: adjuvant-induced arthritis; MTX: methotrexate.

On both days 23 and 44, the SOD and CAT activities and the GSH levels were significantly reduced in the AV group compared to the NV group. On day 44, the AM rats showed a significant decline in their SOD and CAT activities and their GSH levels compared to the AV rats. Nonetheless, on both days 23 and 44, the arthritic treated group (AS) displayed significantly higher SOD and CAT activities and GSH levels compared to the AV and AM groups, with no significant difference compared to the normal vehicle group (NV) on both days 23 and 44. On day 44, the combination group (AMS) showed a significant improvement in its SOD and CAT activities and GSH levels compared to the positive control group (AIA + MTX). On day 44, the AM group showed a significant decline in its SOD and CAT activities and levels of GSH relative to the activities and levels on day 23 (Figure 8A–C).

## 3. Discussion

MTX continues to be used as the first-choice anti-rheumatic drug in RA. Nevertheless, it is associated with several side effects, most notably hepatotoxicity [23,24]. Thus, a combinatorial therapy with MTX is strongly recommended, with the aim of minimizing these limitations. Indeed, due to their long-lasting anti-inflammatory activity with a lower toxicity and fewer side effects, natural compounds have gained much attention in recent years. In this study, in an experimental arthritis model, SIL was selected as a candidate for a combination therapy with MTX. We investigated the possible synergistic effect of SIL with MTX in an AIA rat model. Because SIL resulted in a marked reduction in hepatic damage in rats with methotrexate-induced hepatotoxicity by increasing the antioxidant capacity [25], this research was expanded to investigate the effect of SIL administration on hepatocellular injury caused by MTX in AIA rats.

**Figure 9:**
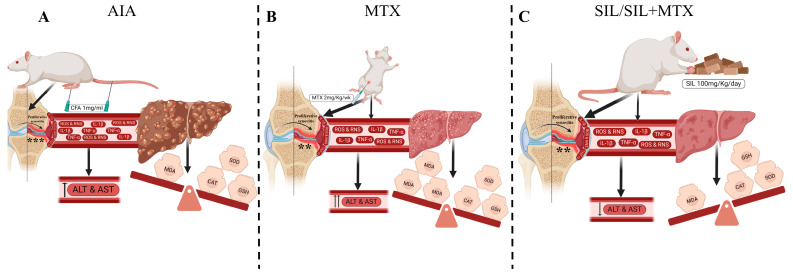
Schematic diagram displaying (**A**) effect of CFA injection on healthy rats (AIA model); (**B**) effect of MTX on AIA rats; and (**C**) effect of SIL or SIL + MTX on AIA rats. CFA: complete Freund’s adjuvant; AIA: adjuvant-induced arthritis; MTX: methotrexate; SIL: silibinin; ALT: alanine aminotransferase; AST: aspartate aminotransferase; CAT: catalase; MDA: malondialdehyde; GSH: reduced glutathione; SOD: superoxide dismutase; ROS: reactive oxygen species; RNS: reactive nitrogen species; ***: synovial hyperplasia, grade 3; **: synovial hyperplasia, grade 2.

Our findings revealed that an injection of 1 mg/mL of CFA induced significant arthritis in the injected paw (Figure 9A), in line with previous studies [26]. The injected paw showed clear swelling in all the arthritic groups. In addition to the presence of proliferative synovitis, significant cellular infiltrates, aggregate formation, and the severe proliferation of destructive layers of granulation tissue were observed. However, the arthritic untreated rats showed systemic inflammation with a higher ESR and a decrease in body weight.

A common finding associated with adjuvant-induced arthritis is liver dysfunction [27]. In our study, an increased level of hepatospecific serum markers (ALT and AST) were associated with CFA-induced liver impairment (Figure 9A). Moreover, in this study, we demonstrated that the AIA model is associated with a significant increase in an oxidative stress biomarker (TBARS) and also a significant decrease in the status of the antioxidant defense system (SOD, CAT, and GSH) in the liver (Figure 9A). Our results support the findings of other studies showing that oxidative stress plays an important role in AIA pathogenesis [28]. In addition to the occurrence of portal and lobular inflammation with central vein obstruction, the histopathological examination showed a parenchymal alteration in the liver, indicating liver toxicity. These findings are consistent with other research examining the action of CFA-induced arthritis in rats [29,30,31,32,33,34].

The arthritic rats were treated with SIL and/or MTX. MTX was given at a dose of 2 mg/kg/week, which mimicked the therapeutic dose used to treat RA in patients (~22.5 mg/week) [35,36]. Hepatotoxicity is known to occur in RA patients receiving a long-term administration of the therapeutic dose of MTX [13,37,38,39]. In addition, our findings, like other published data [40,41], clearly demonstrated the harmful effect of MTX treatments on the liver (Figure 9B). As indicated by the elevated hepatospecific serum markers (ALT and AST) and supported by the histopathological test, it was evident that the MTX administration intensified the alteration of the liver parenchyma (Figure 9B). Methotrexate (MTX) is known to have adverse effects on the liver’s antioxidant defense mechanism. It can increase the production of toxic by-products such as reactive oxygen species (ROS) and inhibit the cofactors of several antioxidant enzymes [42,43,44]. This reduction in the activities of protective antioxidant enzymes such as SOD, CAT, and non-enzymatic GSH can cause a surge in lipid peroxidation (Figure 9B). SOD is considered to be the most effective antioxidant enzyme [45], and it acts as the first line of defense against the harmful effects of molecular oxygen radicals in cells. It is worth noting that the levels of SOD were not affected by MTX injections on day 23 in this study. This suggests that prolonged oxidative stress may be required to trigger an increase in SOD activity. However, chronic MTX treatments may lead to reduced antioxidant defense over time [46]. However, after six injections of MTX (day 44), the SOD levels clearly decreased in the arthritic positive control group receiving MTX (AM group). As a preventive antioxidant, catalase plays an important role in protecting against the deleterious effects of lipid peroxidation [47]. In the present study, the catalase activity increased after 3 weeks of MTX administration, and then it decreased significantly on week 6 when compared to the arthritic untreated group (AV), suggesting that peroxidative damage to tissues occurred in response to the prolonged treatment with MTX. Glutathione (GSH) is one of the main endogenous antioxidants in cells and is involved in diverse functions [48]. The present study showed a highly significant reduction in the cellular GSH levels after the MTX treatment, indicating an imbalance between antioxidants and pro-oxidants in the liver (Figure 9B). A recent study reported that GSH can be involved in the enzymatic detoxification reaction of ROS as a cofactor or as a coenzyme [49,50].

SIL is a constituent of Silybum marianum, which is pharmacologically active. SIL has important antioxidant, anticancer, and anti-inflammatory properties [14,51]. Dupuis et al. demonstrated an immunosuppressive role of silibinin in RA patients, supporting its application in the treatment of autoimmune diseases [52]. In our study, 100 mg/kg of SIL was administered to arthritic rats through their diet, which is considered therapeutic and safe [53,54]. SIL markedly attenuated the progression and severity of adjuvant-induced arthritis in rats (Figure 9C). SIL diminished the inflammatory process, whereby arthritic rats treated with SIL exhibited decreased damage to the joint, which was evident macroscopically (macroscopic scoring) and microscopically (H&E staining). Moreover, the deleterious systemic effects triggered by arthritis were alleviated upon treatment with SIL. SIL decreased the weight loss that was caused by arthritis. The SIL-treated groups exhibited normal ALT and AST liver enzymes. In addition, SIL significantly decreased AIA- and MTX-induced portal/lobular inflammation and hepatocyte degeneration (Figure 9C). Other researchers have reported that SIL ameliorates hepatotoxicity through a reduction in the serum levels of ALT and AST enzymes and through the alleviation of liver pathology [55,56,57]. SIL has long been known for its ability to protect vital liver functions [58,59,60]. Furthermore, SIL has been reported to inhibit the production of inflammatory cytokines and induce apoptosis in RA-pathogenesis-related cells and in a CIA animal model, confirming the therapeutic function of SIL [61].

SIL’s antioxidant activity tends to be its main cellular mechanism [62]. SIL is reported to be an excellent protective agent for cell membranes against lipid peroxidation [63]. In this study, SIL significantly reduced an oxidative stress marker (MDA) in arthritic rats, with a persistent decline observed up to the 44th day (Figure 9C). Furthermore, SIL, when combined with MTX, significantly reduced the elevated MDA levels—increased by MTX—with a prolonged effect being observed on day 44. Previous studies have reported the protective role of SIL through the scavenging of free radicals and antioxidants [64]. SIL also restored the SOD activity. Accordingly, the co-administration of SIL with MTX injections increased the SOD levels, relieving the oxidative stress in comparison to the group receiving MTX alone. The antioxidant and hepatoprotective effect of SIL was also evident by increasing both the catalase activity and the GSH levels in arthritic treated rats (AS). The co-administration of SIL was effective, with a prolonged effect on day 44. Kalemci et al. reported that SIL attenuated MTX-induced pulmonary injury by relieving oxidative stress [65].

The results of this preclinical study provide evidence on the therapeutic benefits of SIL compared to the commonly used anti-rheumatic drug MTX, suggesting promising effects for its use in the treatment of RA. SIL was able to markedly down-regulate the liver injury induced by AIA and MTX, as indicated by the decreased levels of liver function enzyme levels (ALT and AST), which was confirmed by histological examination. In addition, SIL clearly eased the oxidative stress induced by AIA and MTX in the liver by decreasing the levels of lipid peroxidation (MDA) and increasing the antioxidant defense system (GSH, catalase, and SOD).

## 4. Materials and Methods

### 4.1. Chemicals and Reagents Used

Complete Freund’s adjuvant was purchased from InvivoGen, San Diego, CA, USA (vac-cfa-10). Methotrexate (≥98% (HPLC), A6770); silibinin (containing both A and B diastereomers, ≥98% (HPLC), S0417); DTNB (≥98%, D-8130); NADPH (≥97% (HPLC), N-7505); glutathione (GSH ≥ 98.0%, G4251); glutathione reductase; polyvinylpolypyrrolidone (PVPP, 77627); and nitrotetrazolium bluechloride (NBT, ≥90.0% (HPLC), 298-83-9) were purchased from (Sigma-Aldrich Co, St. Louis, MA, USA). Alanine aminotransferase (ALT) kits were purchased from Analyticon ^®^ Biotechnologies AG, Frankfurt, Germany. Aspartate aminotransferase (AST) kits were purchased from Spinreact, S.A./S.A. U Ctra. Santa Coloma, Gerona, Spain. Trichloroacetic (TCA); thiobarbituric acid (TBA)*;* hydrogen peroxide, (H2O2 30% *w*/*v*); L-methionine (149 g/mol); riboflavin (376 g/mol); potassium dihydrogen orthophosphate (KH2PO4); dipotassium hydrogen orthophosphate (K2HPO4); and EDTA sodium salt were made available by the Beirut Arab University.

### 4.2. Animals

For the induction of AIA rat models, male Sprague Dawley rats with an age of four to six weeks (180–220 g) were used. The rats were housed under a 12 h light/12 h dark cycle with free access to a regular pellet diet and water ad libitum under constant conditions at 22 ± 2 °C. Before the experiments began, the rats were left to acclimate for a week. Animal safety and all studies were conducted according to the institutional review board (IRB) requirements established by the Beirut Arab University and approved by the institutional review board of the Beirut Arab University, Lebanon (approval code: 2017A-0030-S-P-0214).

### 4.3. Induction of AIA Model

The male Sprague Dawley rats were injected with CFA containing heat-killed Mycobacterium tuberculosis (strain H37Ra) (1 mg/mL) according to the protocol followed by Darwish, S.F., et al. [31]. A subcutaneous injection of 0.1 mL of CFA at a concentration of 1 mg/mL was administered in the sub-plantar layer of the left hind paw. A booster injection of 0.1 mL was given subcutaneously into the tail on the same day and also on the following day. 

### 4.4. Drug Doses and Preparations

The dose used for SIL in rats reflected the safe dose used in humans (100 mg/kg/day given in the diet) [53,54]. A total of 100 mg of SIL was dissolved in 3% of a carboxy methyl cellulose (CMC) solution. The dose chosen for MTX reflected the therapeutic dose used to treat human RA (2 mg/kg/week given intraperitoneally) [24,66]. A total of 3 mg of methotrexate was dissolved in 10% DMSO.

### 4.5. Timeline and Study Design

**Figure 10:**
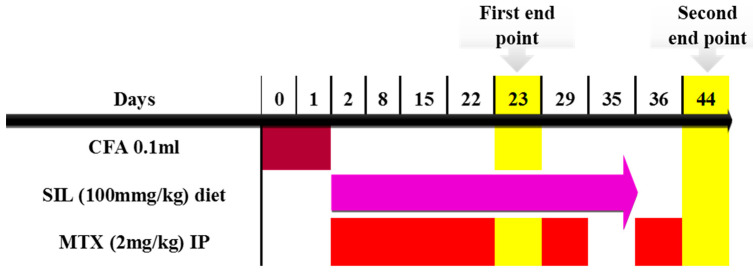
Timeline and design used for the study. CFA: complete Freund’s adjuvant; MTX: methotrexate; SIL: silibinin.

The induction of arthritis was performed on day 0 and day 1. Treatment with SIL and MTX began on day 2. Crackers were soaked in the SIL solution and provided in the diet daily for 33 days, up to day 35. However, MTX was given once per week for 6 weeks until day 44. The timeline of the experimental design included two endpoints that served multiple purposes. The first endpoint was used to test the anti-inflammatory effect of SIL on the AIA rats. This required the experiment to be terminated at no later than 25 days, as the inflammation induced in the AIA model would subside, as described by Chondrex Inc, Redmond, WA, USA. Therefore, half of the rats were euthanized on day 23, which was the first endpoint of the study. The second endpoint was important for assessing the antioxidant activity of SIL against the prolonged use of MTX. Hence, the second half of the rats were euthanized on day 44. Additionally, this study aimed to test if the discontinuation of the treatment would offer a prolonged hepatoprotective effect against the continued weekly injections of methotrexate. For this purpose, the treatment with SIL was discontinued on day 35, while the MTX treatment continued until day 44 (Figure 10). Tissues and blood samples were collected at the two end points.

### 4.6. Experimental Groups

A total of 56 male Sprague Dawley rats were divided into 7 groups (n = 8). (I) Normal group (N): the rats received no drug or treatment of any sort; (II) normal + vehicle group (NV): the normal rats received 3% CMC in their diet; (III) arthritic group (AIA): the rats received 0.3 mL of CFA (model group); (IV) AIA + vehicle (AV): the rats received 0.3 mL of CFA + 3% CMC in their diet, which served to establish if the vehicle possessed any added effects (negative control); (V) AIA + MTX group (AM): the rats received 0.3 mL of CFA with 2 mg/kg/week of methotrexate intraperitoneally (positive control); (VI) AIA + SIL group (AS): the rats received 0.3 mL of CFA with 100 mg/kg/day of SIL in their diet (treated group); and (VII) AIA + SIL + MTX group (AMS): the rats received 0.3 mL of CFA with 2 mg/kg/week of methotrexate intraperitoneally and 100 mg/kg/day of SIL in their diet (combination group).

### 4.7. Experimental Model Assessment

#### 4.7.1. Body Weight Measurement

On days 2, 8, 16, and 22 (once per week), the rats’ body weight was monitored. The difference in body weight was calculated between day 2 and day 22.

#### 4.7.2. Macroscopic Assessment of the Injected Paw

The swelling and inflammation of the hind paw injected with CFA were monitored and evaluated once a week on days 2, 8, 16, and 22 to observe the progression of the disease [24]. A macroscopic scoring system was used to evaluate inflammation, erythema, and deformities, with a range from 0 to 4 [32,67]. The scoring system was as follows: 0 indicated no inflammation or erythema; 1 indicated mild redness and swelling, either in the ankle or wrist, or apparent redness and swelling in individual digits; 2 indicated modest inflammation and erythema; 3 indicated severe inflammation and erythema; and 4 indicated total ankylosis and the failure to extend the knee or wrist.

#### 4.7.3. Tiobiotarsal Joint Histology

A histological examination of the ankle’s tibiotarsal joint was carried out using H&E staining to evaluate the pannus development, cellular infiltration, and synovial hyperplasia [29]. The left hind paws were removed, preserved in 10% neutral-buffered formalin, and then demineralized for two weeks in a solution containing 8% hydrochloric acid and 10% formic acid. The ankle joints were stained with H&E after being transected in the sagittal plane [68]. A pathologist who was blind to the treatment graded the pathological alterations on a scale of 0 to 3.

#### 4.7.4. ESR: Erythrocyte Sedimentation Rate Determination

The erythrocyte sedimentation rate was determined by the Westergren method to monitor the progression of inflammation [66]. On days 23 and 44, whole blood was withdrawn from the rats. An amount of 0.8 mL of blood was mixed with a solution of 0.2 mL of 3.8% sodium citrate (blood:sodium citrate ratio of 1:4). The sample was gently inverted several times and slowly sucked into the Westergren tube to the zero mark. The tubes were mounted vertically and the ESR was read as clear plasma mm 1 h later.

### 4.8. Biochemical Assessment

The rats were sacrificed on days 23 and 44 using anesthesia and cervical dislocation. Blood from the heart was collected and the liver was isolated and used for biochemical and histological evaluations.

#### 4.8.1. Hepatic Enzymes

Serum alanine aminotransferase (ALT) and aspartate aminotransferase (AST) were measured according to the kits’ standard protocol.

#### 4.8.2. Lipid Peroxidation Assay (MDA)

To determine the level of liver lipid peroxidation, we measured the amount of MDA that reacted with thiobarbituric acid to create a pink complex with two TBA molecules. This chromogenic adduct was quantified at 532 nm [69]. Calculated as TBARS, the content of MDA in the liver homogenate was expressed in terms of nmol/mg of protein.

#### 4.8.3. Superoxide Dismutase Assay (SOD)

The SOD activity assay was based on inhibiting the reduction of NBT to formazan by superoxide radicals. The reduction was measured at 560 nm [70]. The SOD activity was expressed in units/mg of protein in the liver homogenate.

#### 4.8.4. Catalase Assay (CAT)

A spectrophotometric procedure measuring peroxide removal was used to measure catalase activity. Spectrophotometrically at 240 nm, the absence of peroxide was tracked. [71]. The CAT activity was expressed in mmol/min/mg of protein in the liver homogenate.

#### 4.8.5. Reduced Glutathione Assay (GSH)

This experiment is based on GSH’s reaction to DTNB (also known as Ellman’s reagent), which generates the 412 nm total absorbent TNB chromophore and oxidized glutathione–TNB adduct (GS–TNB). The frequency of TNB formation, calculated at 412 nm, is proportional to the sample’s GSH concentration [72]. The GSH content was expressed in nM/mg of protein in the liver homogenate.

### 4.9. Liver Histology

A section of the liver was excised and placed into 10% neutral-buffered formalin. After 48 h in the fixative solution, the specimens were processed according to standard methods [68] for H&E staining. The severity of the hepatic injury was measured according to the extent of the following: (1) inflammatory cell infiltration, (2) congested portal vein, (3) distortion in the hepatic chords, and (4) fibrosis and necrosis.

### 4.10. Protein Quantification

The total protein content was determined by Bradford’s method [73]. The protein concentration in the liver homogenate was expressed in mg/mL.

### 4.11. Statistical Analysis

The data collected are presented as the means ± standard deviations. The difference between the groups was statistically evaluated using a variance analysis followed by Tukey’s post-hoc test for the one-way variance analysis and Bonferroni’s adjustment for the two-way variance analysis. The significance level was set at *p* < 0.05. The statistical analysis was performed using GraphPad Prism version 8.0.2 software (San Diego GraphPad Software).

## 5. Limitations of this Study

This study was performed on Sprague Dawley rats rather than on Lewis or Wistar rats, which are extremely susceptible to AIA, due to availability issues. Additionally, the anti-inflammatory and antioxidant effects were not investigated at the molecular level.

## 6. Future Recommendations

Studying the mechanism of action of silibinin at the molecular level is of great interest due to its potent antioxidant and anti-inflammatory effects.

## Data Availability

The datasets analyzed during the current study are available from the corresponding author upon reasonable request.
